# Neurogranin and YKL-40: independent markers of synaptic degeneration and neuroinflammation in Alzheimer’s disease

**DOI:** 10.1186/s13195-015-0161-y

**Published:** 2015-12-24

**Authors:** Konstantin Hellwig, Hlin Kvartsberg, Erik Portelius, Ulf Andreasson, Timo Jan Oberstein, Piotr Lewczuk, Kaj Blennow, Johannes Kornhuber, Juan Manuel Maler, Henrik Zetterberg, Philipp Spitzer

**Affiliations:** Department of Psychiatry and Psychotherapy, University clinic Erlangen and Friedrich-Alexander University Erlangen-Nürnberg, Schwabachanlage 6, 91054 Erlangen, Germany; Clinical Neurochemistry Laboratory, Institute of Neuroscience and Physiology, Department of Psychiatry and Neurochemistry, Sahlgrenska Academy at the University of Gothenburg, Mölndal, Sweden; AlzeCure Foundation, Karolinska Institutet Science Park, Huddinge, Sweden; Department of Molecular Neuroscience, UCL Institute of Neurology, London, WC1N 3BG UK; Department of Neurodegeneration Diagnostics, Medical University of Białystok, Białystok, Poland

## Abstract

**Introduction:**

Neuroinflammation and synaptic degeneration are major neuropathological hallmarks in Alzheimer’s disease (AD). Neurogranin and YKL-40 in cerebrospinal fluid (CSF) are newly discovered markers indicating synaptic damage and microglial activation, respectively.

**Methods:**

CSF samples from 95 individuals including 39 patients with AD dementia (AD-D), 13 with mild cognitive impairment (MCI) due to AD (MCI-AD), 29 with MCI not due to AD (MCI-o) and 14 patients with non-AD dementias (non-AD-D) were analyzed for neurogranin and YKL-40.

**Results:**

Patients with dementia or MCI due to AD showed elevated levels of CSF neurogranin (*p* < 0.001 for AD-D and *p* < 0.05 for MCI-AD) and YKL-40 (*p* < 0.05 for AD-D and *p* = 0.15 for MCI-AD) compared to mildly cognitively impaired subjects not diagnosed with AD. CSF levels of neurogranin and YKL-40 did not differ between MCI not due to AD and non-AD dementias. In AD subjects no correlation between YKL-40 and neurogranin was found. The CSF neurogranin levels correlated moderately with tau and p-tau but not with Aβ_42_ or the MMSE in AD samples. No relevant associations between YKL-40 and MMSE or the core AD biomarkers, Aβ_42_, t-tau and p-tau were found in AD subjects.

**Conclusions:**

Neurogranin and YKL-40 are promising AD biomarkers, independent of and complementary to the established core AD biomarkers, reflecting additional pathological changes in the course of AD.

**Electronic supplementary material:**

The online version of this article (doi:10.1186/s13195-015-0161-y) contains supplementary material, which is available to authorized users.

## Introduction

Alzheimer’s disease (AD) is the most prevalent neurodegenerative disorder worldwide. The major pathological hallmarks of AD include extracellular depositions of β-amyloid (Aβ) peptides as well as intracellular neurofibrillary tangles consisting of hyperphosphorylated tau, loss of synapses, and neuroinflammation [1, 2]. The earliest pathophysiological events are expected to occur 10–20 years before the onset of dementia [3]. Changes in cerebrospinal fluid (CSF) biomarkers reflecting amyloid pathology (Aβ_42_) and neurodegeneration [total tau (t-tau) and phosphorylated tau (p-tau)] occur early in the course of AD and are increasingly implicated in the early and predictive diagnosis of AD [4, 5]. The accuracy of diagnosis based on these core AD biomarkers is high, as long as markers of neurodegeneration and amyloidosis are altered concordantly [6]. However, in a proportion of patients, biomarker results may be contradictory, leading to lower diagnostic accuracy [7]. Additionally, Aβ_42_, t-tau, and p-tau allow no conclusions about cognitive performance and only a limited prediction of cognitive decline to be made, a feature that is especially important for clinical trials [8]. Therefore, additional biomarkers reflecting further aspects of AD pathophysiology, such as synaptic degeneration and neuroinflammation, are needed. Loss of synapses is an early event in the course of AD, and the correlation between synapse density and performance on neuropsychiatric tests such as the Mini Mental State Examination (MMSE) and verbal fluency tests is well established [9–12]. Neurogranin is a postsynaptic protein expressed in the neocortex, amygdala, caudate nucleus, putamen and hippocampus in the rodent brain [13]. In the human brain, expression is highest in associative cortical areas [14], suggesting a link with cognition. It is concentrated in dendritic spines of principal excitatory synapses, and its translocation to dendritic spines is impaired in AD [15–17]. Neurogranin levels are reduced in the hippocampus and cortex in AD, indicating a loss of dendrites [2].

Synaptic proteins, including neurogranin, have been shown to be present in the CSF [18]. A first pilot study using immunoprecipitation (IP) and Western blot analysis showed a marked increase in CSF neurogranin levels in AD [19]. In a later study, using both IP-mass spectrometry and a newly developed immuno-based assay, researchers verified elevated levels of CSF neurogranin in a larger cohort of patients with AD [20, 21]. Importantly, high CSF neurogranin levels were also found in prodromal AD cases, and the degree of increase correlated with the rate of future cognitive decline [21].

Neuroinflammation is another common feature of AD pathology, and several epidemiological studies suggest a decrease in risk for AD after long-term administration of nonsteroidal anti-inflammatory drugs [22]. YKL-40, a 39 kDa glycoprotein homologue to chitinase, is a marker for macrophage and microglial differentiation and activation [23–25]. Elevated CSF levels were shown in several infectious and noninfectious disorders of the central nervous system (CNS) [26]. Also, in AD, YKL-40 seems to be elevated in CSF [27–29]. The aim of this study was to investigate whether neurogranin as a marker for synaptic loss reflects cognitive disturbances and, together with YKL-40, shows aspects of AD pathophysiology complementary to amyloid pathology and neurodegeneration.

## Methods

### Patients and sample collection

The study protocol was approved by the ethics committee of the university clinic Erlangen-Nürnberg (number 3987), and all participants provided written informed consent. The patients were recruited in the memory clinic of the Department of Psychiatry and Psychotherapy in Erlangen, Germany. The participants underwent a physical, neurological, psychiatric, and neuropsychological examinations according to the Consortium to Establish a Registry for Alzheimer’s Disease test battery [30]. Clinical diagnosis was supported by a brain magnetic resonance imaging scan, hexamethylpropyleneamine oxime single-photon emission computed tomography, and positive CSF biomarkers. Neurochemical dementia diagnosis was made using certified enzyme-linked immunosorbent assay (ELISA) kits for Aβ_1-40_ (IBL International, Hamburg, Germany), Aβ_1-42_ (The Genetics Company, Schlieren, Switzerland; and IBL International), and t-tau and p-tau (Fujirebio, Gent, Belgium; and IBL International). Diagnoses of AD and mild cognitive impairment (MCI) were made according to the revised National Institute on Aging–Alzheimer’s Association (NIA-AA) criteria [4, 5]. None of the patients had a history indicative of hereditary AD. Subjects with malignant diseases or signs of systemic inflammation were excluded.

Experienced physicians collected the CSF samples by lumbar puncture in the L3-L4 or L4-L5 intervertebral space. With the exception that samples were centrifuged at 1500 × *g* instead of 2000 × *g*, sampling and storage were carried out according to international consensus guidelines within 90 minutes after sampling [31].

### Neurogranin assay

Measurement of neurogranin on the Meso Scale Discovery (MSD; Rockville, MD, USA) platform was performed as described previously [21]. The in-house monoclonal mouse antibody Ng7, which binds amino acids 52–65 of neurogranin, was used on a QUICKPLEX 96-well plate (MSD) as the capturing antibody. After blocking with 5 % MSD Blocker for 1 h at room temperature (RT), the full-length neurogranin calibrators in concentrations ranging from 31.3 pg/ml to 4000 pg/ml, the blanks, and 50 μl of CSF sample for each well were added in duplicates and coincubated overnight with a polyclonal anti-neurogranin antibody (ab 23570; EMD Millipore, Billerica, MA, USA). The next day, the plates were washed and SULFO-TAG goat anti-rabbit antibody (MSD) (25 μl/well) was added for 2 h at RT. Before the plates were read on a QUICKPLEX SQ 120 reader (MSD), 150 μl of 2× MSD read buffer with surfactant (MSD) was added to the wells. The samples were analyzed without knowledge of the clinical diagnosis. Intra-assay variation of the assay was calculated as the median of range/average from duplicate measurements, and the result was 10.4 %. Measurements of samples with a coefficient of variation (CV) above 20 % were repeated. The interassay CV was 14.2 %, as indicated by positive controls that were run on every plate. The detection ranges were 57.5–4000 pg/ml on the first plate and 69.4–4000 pg/ml on the second plate.

### YKL-40 assay

YKL-40 levels were measured with a commercially available ELISA kit (R&D Systems, Minneapolis, MN, USA) according to the manufacturer’s instructions. This assay has been validated previously for CSF [28, 29, 32, 33]. For the YKL-40 analyses, the CSF was diluted 1:100. The samples were analyzed without knowledge of the clinical diagnosis. Intraassay CVs were all below 10 %. The interassay CV, as indicated by positive controls run on every plate, was 5.6 %.

### Statistical analysis

The statistical analyses were performed with GraphPad Prism 6 software (GraphPad Software, La Jolla, CA, USA). Because data were skewed, group comparisons were made using the nonparametric Kruskal–Wallis test followed by Dunn’s posttest. Correlations were determined using Spearman’s rank correlation coefficient. Receiver operating characteristic (ROC) curves were drawn by plotting the false-positive fraction (100 % − specificity) versus the true-positive fraction (sensitivity). A *p* value below 0.05 was considered significant.

## Results

### Patient characteristics

In total, 95 CSF samples collected at the Department of Psychiatry and Psychotherapy of the Universitätsklinikum Erlangen were included in the study and categorized according to the NIA-AA criteria. The cohort consisted of 39 patients with Alzheimer’s disease dementia (AD-D) comprising patients with probable AD dementia with high evidence of AD pathophysiological process, patients with possible AD dementia with high evidence of AD pathophysiological process, and patients with probable AD dementia with intermediate evidence of AD pathophysiological process; 13 patients with MCI with a high likelihood that the mild cognitive impairment was due to Alzheimer’s disease (MCI-AD); 29 patients with mild cognitive impairment unlikely due to Alzheimer’s disease (MCI-o); and 14 patients with dementia unlikely due to AD (non-AD-D) (Table 1). The group of patients with other dementias included seven patients with frontotemporal dementia, three with vascular dementia, one with Lewy body dementia, and three with dementia of unknown origin. As AD biomarkers are applied mostly in the differential diagnosis of cognitive disturbances, MCI-o was chosen as the reference group. This group consisted especially of patients with depression, vascular disease, and early frontotemporal dementia (Table 1). The study population was well balanced overall according to age and sex. A significant difference in age was evident only between the non-AD-D and AD-D cohorts (*p* < 0.05). The core biomarkers Aβ_42_, t-tau, and p-tau differed highly significantly in patients with MCI-AD and patients with AD compared with those in the MCI-o and non-AD-D groups (*p* < 0.001). As expected, MMSE scores in the non-AD-D and AD-D cohorts were significantly lower than in the MCI samples.

**Table 1:** Patient characteristics

	MCI-o	Non-AD-D	MCI-AD	AD-D
Number of patients	29	14	13	39
Age, yr	69.4 [61–75]	65.1 [59–71]	73.3 [69–77]	72.5 [68–76]^a^
Sex, M/F	15/14	6/8	5/8	18/21
MMSE	27 [26–28]	20 [20–23]^b^	26 [25–28]^c^	21 [19–24]^b^
Aβ_1-42_, pg/ml	1262 [1014–1626]	1255 [997–1585]	638 [590–852]	796 [618–928]
Aβ_1-40_, pg/ml	15,393 [12,208–21,112]	13,332 [9303–22,989]	21612 [17,875–26,191]	20,803 [15,168–24,448]
t-tau, pg/ml	226 [158–246]	242 [189–320]	580 [487–789]	522 [403–708]
p-tau, pg/ml	44 [29–59]	46 [41–53]	92 [80–113]	99 [74–111]

*Aβ* β-amyloid, *p-tau* phosphorylated tau, *t-tau* total tau, *MMSE* Mini Mental State Examination, *MCI-o* mild cognitive impairment not due to Alzheimer’s disease, young control subjects without dementia, *non-AD-D* dementia not due to Alzheimer’s disease, *MCI-AD* mild cognitive impairment due to Alzheimer’s disease, *AD-D* Alzheimer’s disease dementia

The values represent the median [interquartile range]

Differences between the groups were calculated using a nonparametric Kruskal-Wallis test followed by Dunn’s posttest. No *p* values were calculated for Aβ, t-tau, and p-tau, as the patients were selected according to these markers

^a^ *p* < 0.05 vs. non-AD-D

^b^ *p* < 0.001 vs. MCI-o

^c^ *p* < 0.01 vs. non-AD-D

### Elevated levels of neurogranin in Alzheimer’s disease

**Fig. 1:**
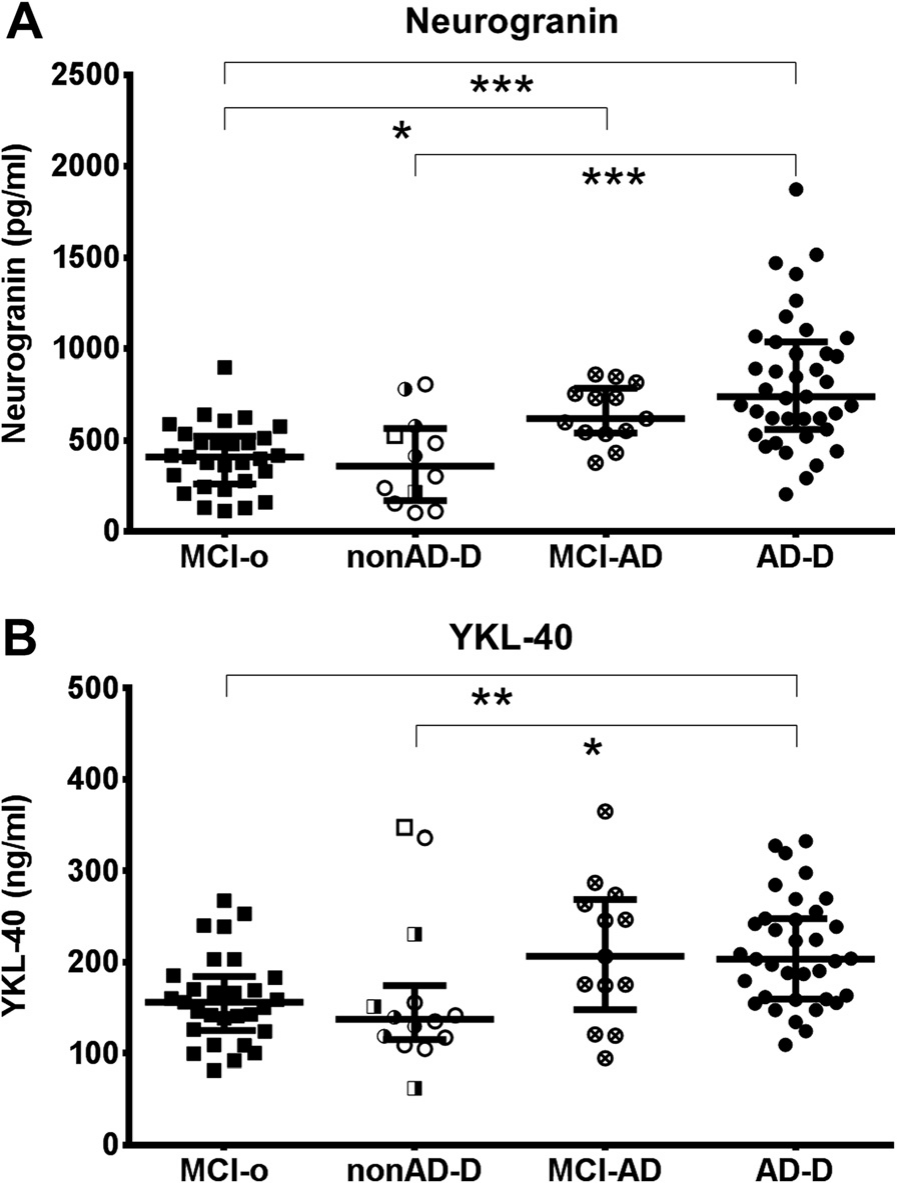
Increased levels of cerebrospinal fluid (CSF) neurogranin and YKL-40 in Alzheimer’s disease. Scatterplots of CSF neurogranin (**a**) and YKL-40 (**b**) in patients with mild cognitive impairment not due to Alzheimer’s disease (MCI-o, black squares), mild cognitive impairment due to AD (MCI-AD, circles with a cross), Alzheimer’s disease dementia (AD-D, black circles), and non-Alzheimer’s disease dementia (non-AD-D), consisting of frontotemporal lobar degeneration (withe circles), vascular dementia (semi-filled squares), dementia with Lewy bodies (white squares), and dementia of unknown origin (semi-filled circles). Data are presented as median and interquartile range. Differences between the groups were calculated with the Kruskal–Wallis test followed by Dunn’s posttest. *p < 0.05, **p < 0.01, ***p < 0.001

Compared with patients with MCI-o, neurogranin was significantly increased in patients diagnosed with MCI-AD (*p* < 0.05) and those with AD dementia (*p* < 0.001). No difference was found between MCI-o and the non-AD-D patients (Fig. 1). The neurogranin levels in MCI-AD patients did not differ from those with suspected AD dementia. No difference in neurogranin levels was found within the different AD dementia populations separated by the certainty of the diagnosis (data not shown).

**Fig. 2:**
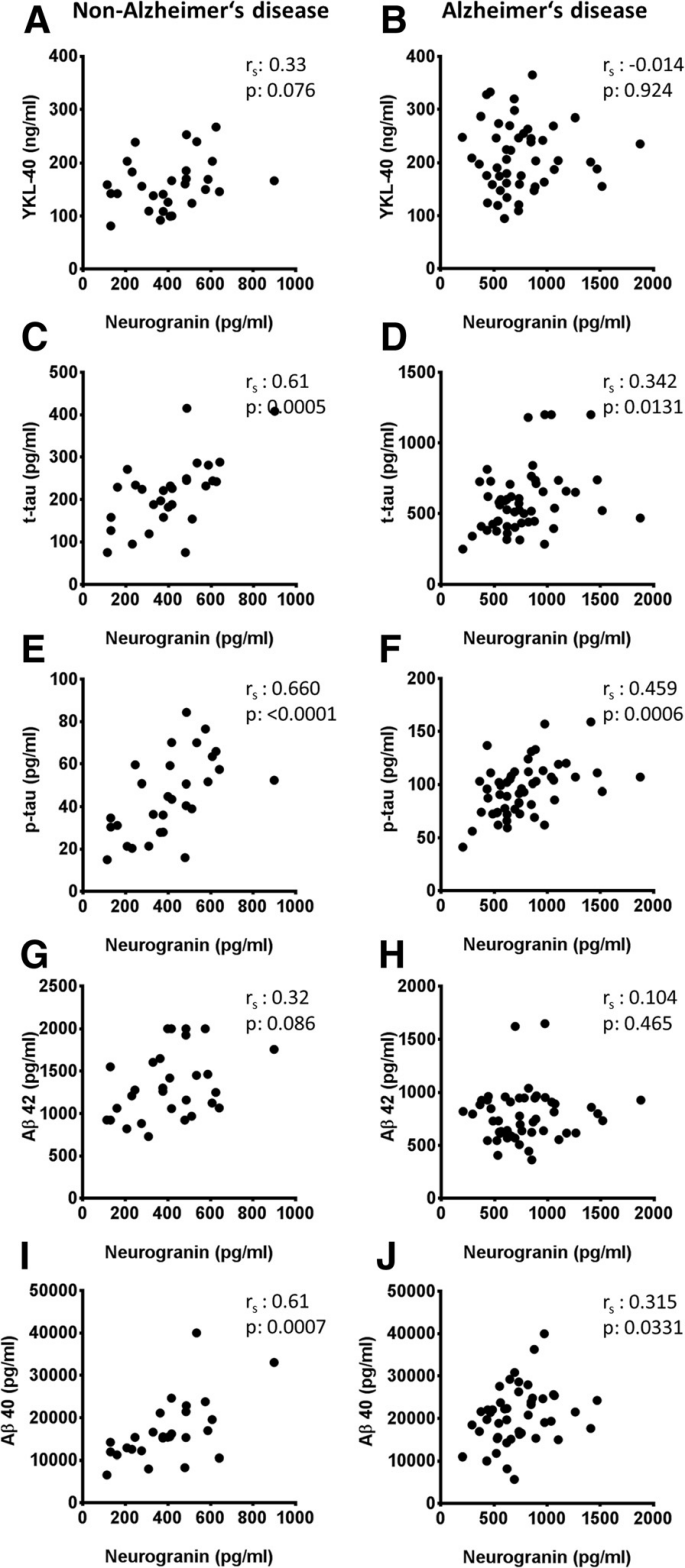
Neurogranin is correlated with total tau (t-tau), phosphorylated tau (p-tau), and β-amyloid (Aβ_40_), especially in subjects without Alzheimer’s disease (non-AD). Cerebrospinal fluid levels of neurogranin in the non-AD group (**a**, **c**, **e**, **g**, and **i**) and patients in the AD group (**b**, **d**, **f**, **h**, and **j**) are plotted against YKL-40 (**a**, **b**) and core AD biomarkers (**c**–**j**). Correlations were calculated using Spearman’s rank correlation coefficient

**Fig. 3:**
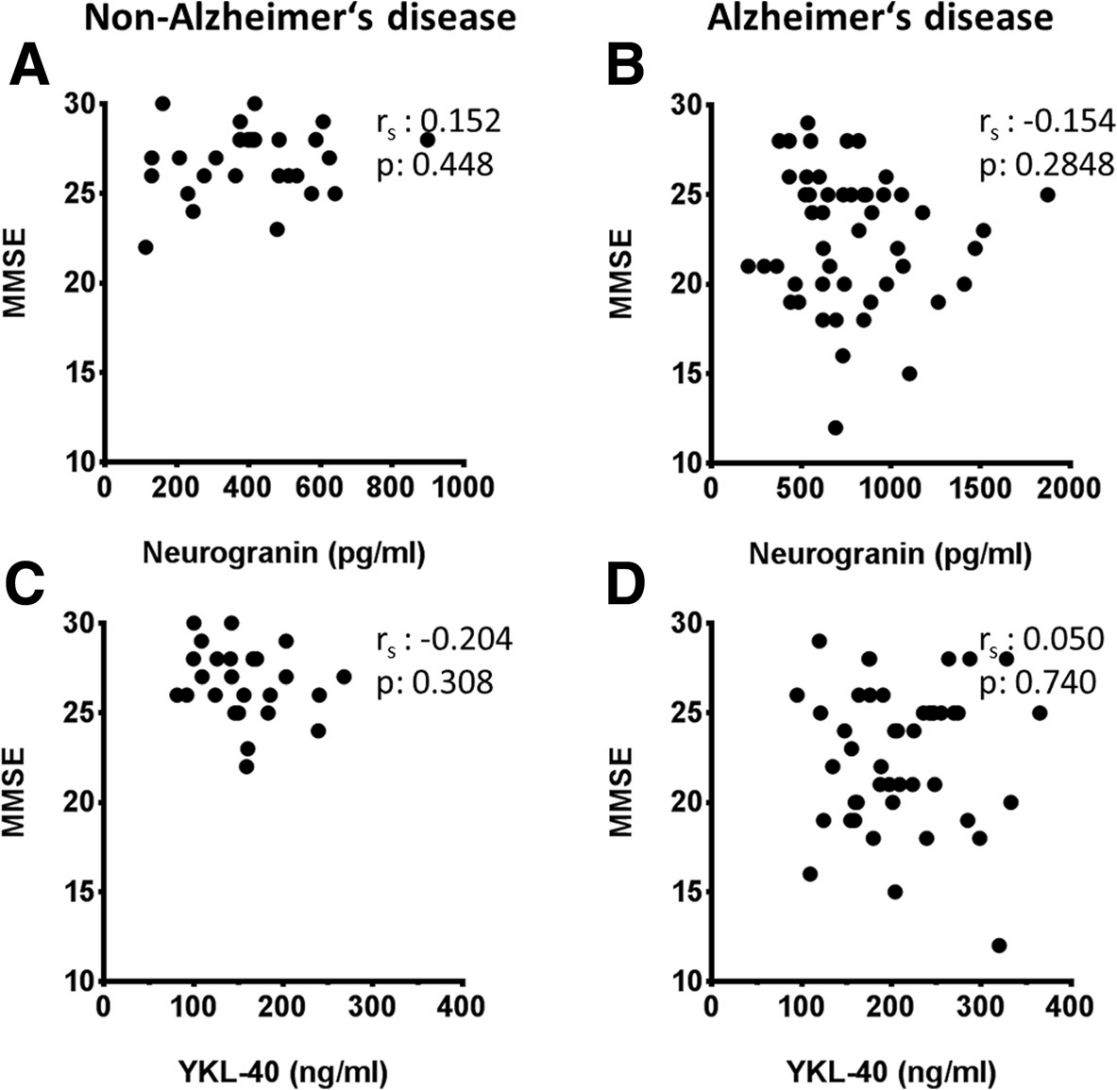
Neurogranin and YKL-40 are not correlated with Mini Mental State Examination (MMSE) scores. Cerebrospinal fluid levels of neurogranin (**a**, **b**) and YKL-40 (**c**, **d**) are plotted against MMSE scores in the non-AD group (**a**, **c**) and the AD group (**b**, **d**). Correlations were calculated using Spearman’s rank correlation coefficient

For the analysis of correlations, MCI-AD and AD-D were merged into an AD group and MCI-o and non-AD-D were merged into a non-AD group. Neurogranin correlated with t-tau and p-tau in the non-AD group and in the AD group (Fig. 2). However, correlations were stronger within the non-AD group (Fig. 2). A correlation between neurogranin and Aβ_1-42_ was found only in the non-AD group (Fig. 2). Interestingly, a strong correlation of neurogranin with Aβ_1-40_ was also found (Fig. 2). The MMSE score did not correlate with neurogranin levels in any of the groups (Fig. 3).

### Elevated levels of YKL-40 in Alzheimer’s disease

CSF YKL-40 levels were significantly elevated in patients with AD dementia as compared with those with MCI-o or non-AD dementia (*p* < 0.05). YKL-40 was also elevated in MCI-AD patients, but without reaching statistical significance (*p* = 0.15). The YKL-40 levels of MCI-AD and AD dementia patients did not differ (Fig. 1). The patients with other forms of dementia did not show an elevation in YKL-40 levels compared with MCI-o patients (Fig. 1).

YKL-40 was age-correlated in our sample (Additional file 1: Figure S1). However, as the populations with cognitive disturbances were age-matched, no statistical correction for age was made. A significant correlation of YKL-40 with t-tau and p-tau was found only in the non-AD group (Additional file 2: Figure S2). In addition, no correlation of YKL-40 with Aβ_1-42_, Aβ_1-40_, or MMSE score was observed (Fig. 3 and Additional file 2: Figure S2).

### No correlation between neurogranin and YKL-40

No significant correlation could be shown between neurogranin and YKL-40, as indicators for postsynaptic integrity and microglial activation in AD (Fig. 2).

### Neurogranin supports the early and differential diagnosis of AD

**Fig. 4:**
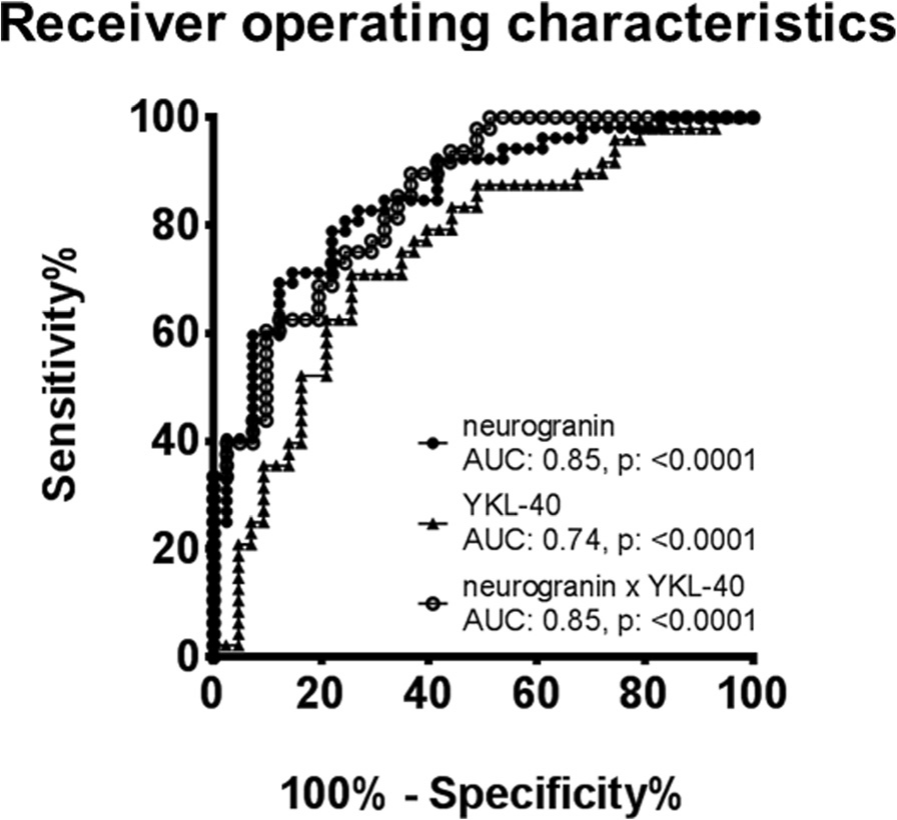
Neurogranin distinguishes Alzheimer’s disease (AD) from non-AD subjects well. Receiver operating characteristic curves of neurogranin (black circles), YKL-40 (black triangles), and the product of neurogranin × YKL-40 (white circles) for the discrimination between samples within the non-AD group and the AD group. *AUC* area under the curve

To test the suitability of neurogranin and YKL-40 as biomarkers for AD, ROC curves were calculated. Samples of patients with AD pathology (AD group) could be separated from those without signs of AD pathology, including other dementias (non-AD group), with an area under the curve (AUC) of 0.85 for neurogranin and 0.66 for YKL-40 (Fig. 4). Combining the two markers by multiplication resulted in an AUC of 0.85 (Fig. 4).

## Discussion

We have shown that the synaptic protein neurogranin and YKL-40 are elevated in the CSF of patients with AD. Even though both markers were significantly increased, they did not correlate with each other in AD.

In the diagnosis of cognitive disturbances, biochemical markers as indicators of the disease are increasingly implicated. Unfortunately, biochemical markers reflecting cognitive decline are still sparse [8]. It has long been known that the number of synapses is well correlated with the degree of cognitive disturbances [10, 34, 35]. Therefore, it is expected that biomarkers indicating synaptic integrity would be well suited to reflect cognitive decline. In our study, CSF neurogranin levels were elevated in AD. However, we found no difference in the levels of neurogranin in the dementia stage versus MCI. In addition, there was no correlation between neurogranin levels and MMSE scores. Thus, our results are in line with previous reports of elevated levels of neurogranin in AD [19, 20, 36, 37]. In contrast to our present study, Thorsell et al. did not distinguish between MCI due to AD and MCI due to other diseases, and they measured neurogranin levels in the MCI group between that of controls and that of patients with AD [19]. In their study, Kvartsberg et al. included a neuropsychological follow-up investigation which showed that high CSF levels of neurogranin at baseline predicted a more rapid decline in cognition [20]. This might indicate that neurogranin reflects not the synaptic density but rather the intensity of current synaptic destruction.

In line with previous studies, we have shown that neurogranin differentiated well between AD and other neurodegenerative diseases. Established core biomarkers (i.e., Aβ_1–42_, t-tau, and p-tau) have high diagnostic accuracy in discriminating individuals with AD from subjects without cognitive disturbances, but their diagnostic performance in differentiating AD from other dementias is far from optimal [38]. Interestingly, CSF neurogranin was not elevated in our cohort of patients with other neurodegenerative diseases. However, the cohort of non-AD-D patients was small and comprised especially patients with frontotemporal lobar degeneration. Further research is necessary to clarify whether the elevation of neurogranin is specific for AD.

The stronger correlation of neurogranin and tau/p-tau in non-AD patients as compared with patients with AD and the missing elevation of neurogranin in non-AD-D patients also points to a mechanism of neurodegeneration in AD distinct from the physiological dying of neurons and distinct from other neurodegenerative diseases. Most likely, it shows a degeneration of synapses that is weakly related to the axonal damage indicated by tau [39]. The exact mechanism by which neurogranin is released is unclear.

Elevated levels of CSF YKL-40 in early stages of AD have been demonstrated in two independent studies, but there are also contradictory data [28, 32, 40]. In our study, we confirmed that YKL-40 is elevated early in the course of AD and that the levels do not change during disease progression. In addition, YKL-40 levels in other dementias did not differ from those with MCI not due to AD. This suggests that neuroinflammation in AD pathology differs from that in other dementias. In accordance with the concept of inflammaging, introduced by Franceschi et al., we found a correlation of YKL-40 with age. *Inflammaging* describes a low-grade, chronic upregulation of inflammatory responses during aging as a risk factor for several age-dependent diseases [41, 42]. Accumulating evidence shows a similar alteration in the CNS of the elderly as a prodrome of AD [43]. In part, this increased immune reactivity in the aged brain might be derived from primed microglia. Primed microglia are in a preactivated state and tend to react in a prolonged manner and by secretion of higher amounts of proinflammatory signals [44]. Excessive inflammatory responses by primed microglia aggravate neurodegeneration, impair synaptic plasticity, and lead to cognitive decline [45]. However, we did not find a correlation between YKL-40 and MMSE. Yet, as a marker for microglial activation, YKL-40 seems well suited to reflect these aspects of AD pathophysiology.

Even though a link between microglial activation and synaptic degeneration can be postulated, we found no correlation between neurogranin and YKL-40 in our study. As detailed above, YKL-40 is a rather unspecific marker that is highly influenced by patients’ comorbidities. This might also explain why data on YKL-40 correlations are somewhat contradictory. Two studies showed a correlation with p-tau and t-tau, whereas a third did not find any correlation with CSF tau levels [27, 29, 40]. Data on correlations with MMSE are likewise conflicting [29, 40]. The missing correlation between neurogranin and YKL-40 suggests that these two markers reflect two different aspects of neurodegeneration in AD. Whereas YKL-40 might represent Aβ-mediated activation of microglia and neuroinflammation, elevated levels of neurogranin might indicate synaptic damage of another origin, such as direct Aβ-mediated neurotoxicity via soluble oligomers, disturbances in calcium homeostasis, or mitochondrial damage [46–52].

To evaluate neurogranin and YKL-40 as potential biomarkers for AD, we determined ROC curves for both markers alone and a combination of both markers by multiplication. With an AUC of 0.85, the diagnostic performance of neurogranin is in the reported range of the isolated core biomarkers. The combination of Aβ, tau, and neurogranin might therefore improve diagnostic performance considerably. A comparison with core biomarkers was not possible in our study, as patients were selected according to these markers. To further evaluate the potential of neurogranin as a diagnostic biomarker, further studies including patients not stratified by established biomarkers are needed. The additional benefit of YKL-40 as biomarker for AD is limited, with an AUC of 0.66, and is a rather unspecific marker. However, YKL-40 might be useful for patient stratification and monitoring of drugs targeting microglial activation.

## Conclusions

Taken together, elevated levels of neurogranin and YKL-40 could be found in CSF samples of patients with AD compared with those with other dementias and control subjects. The expected relationship between postsynaptic damage and microglial activation in AD could not be shown using these markers. Therefore, neurogranin and YKL-40 might support the biochemical dementia diagnosis by reflecting aspects of AD pathophysiology complementary to Aβ and tau.

## Additional files

Additional file 1: Figure S1. YKL-40 is strongly, and neurogranin weakly, correlated with age. CSF levels of neurogranin and YKL-40 are plotted against age in the whole sample. Correlations were calculated with Spearman’s rank correlation coefficient. (TIF 106 kb) Additional file 2: Figure S2. YKL-40 is correlated with t-tau and p-tau in non-AD subjects. CSF levels of YKL-40 in the non-AD group (A, C, E, G) and the AD group (B, D, F, H) are plotted against core AD biomarkers. Correlations were calculated with Spearman’s rank correlation coefficient. (TIF 343 kb)

## Abbreviations

Aβ: β-amyloid
AD: Alzheimer’s disease
AD-D: Alzheimer’s disease dementia
AD group: patients with Alzheimer’s disease (including mild cognitive impairment and dementia)
AUC: Area under the curve
CNS: central nervous system
CSF: cerebrospinal fluid
CV: coefficient of variation
ELISA: enzyme-linked immunosorbent assay
IP: immunoprecipitation
MCI: mild cognitive impairment
MCI-AD: mild cognitive impairment due to Alzheimer’s disease
MCI-o: mild cognitive impairment not due to Alzheimer’s disease (MCI-other)
MMSE: Mini Mental State Examination
MSD: Meso Scale Discovery
NIA-AA: National Institute on Aging–Alzheimer’s Association
non-AD-D: dementia not due to Alzheimer’s disease
non-AD group: patients without Alzheimer’s disease (including mild cognitive impairment and dementia)
p-tau: phosphorylated tau
ROC: receiver operating characteristic
RT: room temperature
t-tau: total tau
