# Supplementary figures and images for: Neurogranin and YKL-40: independent markers of synaptic degeneration and neuroinflammation in Alzheimer’s disease
Source: Alzheimers Res Ther. 2015 Dec 24;7:74. doi: 10.1186/s13195-015-0161-y (PMC4690296; doi:10.1186/s13195-015-0161-y)

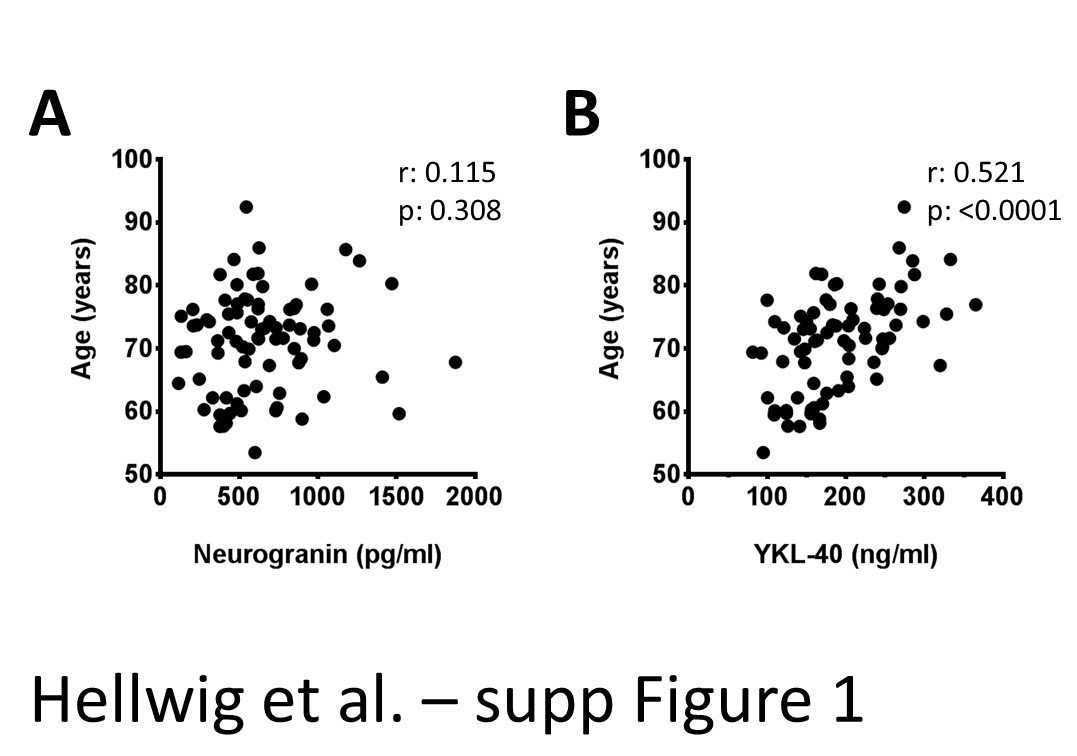

Supplement: Additional file 1: Figure S1. — YKL-40 is strongly, and neurogranin weakly, correlated with age. CSF levels of neurogranin and YKL-40 are plotted against age in the whole sample. Correlations were calculated with Spearman’s rank correlation coefficient. (TIF 106 kb) [file 13195_2015_161_MOESM1_ESM.tif]

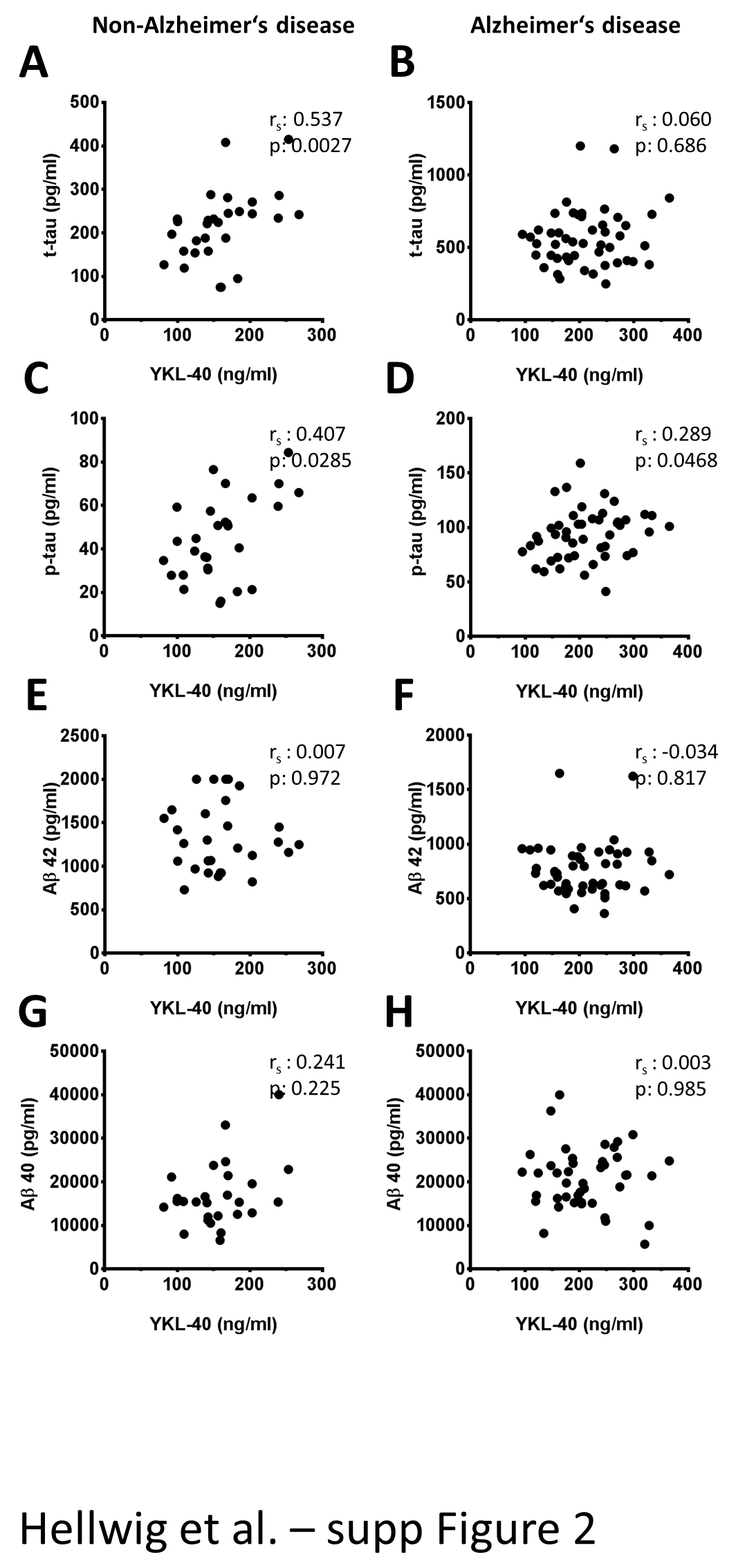

Supplement: Additional file 2: Figure S2. — YKL-40 is correlated with t-tau and p-tau in non-AD subjects. CSF levels of YKL-40 in the non-AD group (A, C, E, G) and the AD group (B, D, F, H) are plotted against core AD biomarkers. Correlations were calculated with Spearman’s rank correlation coefficient. (TIF 343 kb) [file 13195_2015_161_MOESM2_ESM.tif]
